# Compressive Strength and Chloride Resistance of Slag/Metakaolin-Based Ultra-High-Performance Geopolymer Concrete

**DOI:** 10.3390/ma16010181

**Published:** 2022-12-25

**Authors:** Yufei Zhang, Jiejing Chen, Jin Xia

**Affiliations:** 1 Department of Civil and Environmental Engineering, Faculty of Science and Technology, University of Macau, Macao 999078, China; 2 Institute of Structural Engineering, Zhejiang University, Hangzhou 310027, China

**Keywords:** geopolymer concrete, mechanical properties, compressive strength, durability, chloride penetration

## Abstract

Ultra-high performance geopolymer concrete (UHPGC) has been favored due to its excellent sustainability and outstanding mechanical properties. This study was conducted to explore the mechanical and durability properties of slag/metakaolin-based UHPGC with steel fibers reinforcement. The uniaxial compression test and rapid chloride migration test were conducted to measure the compressive strength and chloride penetration resistance of UHPGC. A total of nine groups of mixture proportions were designed and tested to investigate the influences of steel fiber dosage and sodium hydroxide (NaOH) solution concentration. The results showed that an increased steel fiber dosage and alkali concentration can improve compressive strength, and the maximum compressive strength can reach more than 140 MPa. In addition, the rapid chloride migration test showed that the chloride penetration resistance of the slag/metakaolin-based concrete was moderate, with a non-steady chloride migration coefficient ranging from 6.5 × 10^−12^ m^2^/s to 14.1 × 10^−12^ m^2^/s. The increase in steel fiber volume content slightly enlarged chloride penetration depth, while the higher concentration of sodium hydroxide solution was beneficial as it improved chloride penetration resistance. The results suggest that although ultra-high compressive strength can be achieved, the durability issues of steel fiber reinforced slag/metakaolin-based geopolymer concrete still need considerable attention.

## 1. Introduction

The increasing need to improve the performance of concrete materials has resulted in the invention of ultra-high performance concrete (UHPC), which has been favored due to its superior compressive strength, high blast-resistance, excellent abrasion resistance, and good durability [1,2]. However, as a cement-based material, the production of UHPC heavily depends on the consumption of Portland cement [3], and it has been estimated that the production of one ton of clinker would lead to the emission of a nearly equivalent amount of greenhouse gases into the atmosphere [4,5]. Consequently, to ensure the sustainability of construction materials, researchers have attempted to completely substitute Portland cement by using geopolymers to develop geopolymer concrete [6,7,8]. Geopolymers refer to clinker-free binder materials which can be produced via alkaline activation of aluminum silicate sources, such as ground granulated blast furnace slag (GGBFS), metakaolin (MK), and fly ash (FA) [9,10]. The mixed solution of sodium hydroxide and sodium silicate is commonly selected as the alkaline activator solution for alkaline activation or geopolymerization to produce geopolymer concrete [11,12].

In terms of geopolymer concrete, Thomas et al. [13] summarized the production procedure of geopolymer concrete where solid precursors were mixed with alkali solution and aggregated to produce geopolymer concrete with a certain strengths grade. Generally, it can be classified as a unitary system with only one raw material or a binary system with two blended precursors, such as slag and metakaolin or slag and fly ash [14,15]. Previous studies have reported that the adoption of a single precursor, such as slag, may lead to the appearance of efflorescence and excessive shrinkage of geopolymer concrete [16,17]. As a result, researchers have attempted to replace single raw materials with other precursors. Puligilla and Mondal [18] found that slag is beneficial for the property improvement of fly ash-based geopolymer concrete. In addition, because of the higher content of aluminum and its better stability, metakaolin has been used with slag to produce slag/metakaolin based geopolymer concrete with better chemical stability and a relatively lower cost. Hasnaoui et al. [19] reported that using the blended precursors containing metakaolin and ground granulated blast furnace slag can lead to better workability and higher strength compared to slag-based geopolymer concrete. Peng et al.’s [20,21] study showed that the mass ratio between slag and metakaolin of 40%/60% can facilitate the geopolymerization reaction. Chang et al. [22] has also reported that the addition of metakaolin was helpful as it improved chloride binding capacity; hence, it also improved chloride penetration resistance.

In recent years, ultra-high performance geopolymer concrete has been proposed by adding steel fibers into geopolymer concrete, which can reach comparable and even better properties compared to those of UHPC [23,24]. Liu et al. [25] studied the influence of steel fiber dosage and geometry on slag/fly ash based UHPGC, and the results showed that increasing fiber dosage and reducing fiber diameter were both beneficial regarding compressive strength improvement. Wetzel and Middendorf [23] successfully designed slag/metakaolin-based UHPGC with a compressive strength of up to 150 MPa by adding silica fume. Kathirvel and Sreekumaran [26] also reported that replacing slag with silica fume and increasing fiber content can improve the compressive strength of UHPGC. In addition to the influence of raw materials and fiber content, Mousavinejad and Sammak [27] also studied the influence of sodium hydroxide solution concentration on the fracture properties of UHPGC, and the results indicated that increasing the molarity of sodium hydroxide solution within a certain range is helpful to improve the mechanical performance of UHPGC. However, an excessively higher alkali concentration would lead to the efflorescence and shrinkage of geopolymer concrete.

Contrary to the extensive studies focusing on mechanical properties, durability issues of UHPGC have received relatively less attention. Researchers have studied the chloride resistance of UHPGC since chloride is a major deleterious ionic species that can cause corrosion in embedded steel reinforcement [28,29,30,31]. Ambily [32] suggested that slag-based UHPGC possessed higher chloride permeability compared to UHPC. Qidi et al. [33] also mentioned that the corrosion of steel fibers would facilitate chloride transport. More recently, Mousavinejad and Sammak [34] comprehensively studied the chloride resistance of slag-based UHPGC containing steel and polypropylene fibers, and the results showed that the intrusion of fibers in concrete matrix can increase chloride penetration resistance.

As summarized in the literature review, remarkable progresses have been made regarding the mechanical properties of UHPGC, and various influence factors, such as fiber dosage, fiber type, and alkali molarity, have been investigated in previous research. However, most of the studied materials were based on single-phase precursors, such as slag and fly ash, while the mechanical performance of slag/metakaolin-based UHPGC were seldom reported. In addition, although the durability aspects of UHPGC have attracted attention in academia, the research on both the mechanical and durability performances of UHPGC, especially for slag/metakaolin-based UHPGC, is inadequate, and understandings of different influence factors on both aspects are also insufficient.

Regarding the abovementioned knowledge gap, an experimental study concerning both the mechanical and durability properties of slag/metakaolin-based UHPGC was conducted. Firstly, the influence of steel fiber dosage and molarity of sodium hydroxide solution on the compressive strength of UHPGC was investigated. A total of nine groups of specimens with three different fiber volume contents (0%, 1%, and 2%) and NaOH solution concentrations (4 mol/L, 8 mol/L, and 12 mol/L) were designed. Then, a rapid chloride migration (RCM) test was conducted to determine and compare the chloride penetration resistance of UHPGC with different fiber dosages and NaOH solution concentrations in the alkali activator solution. Summaries and conclusions derived from the obtained test results are provided after the methods and results discussions.

## 2. Experimental Program

### 2.1. Raw Materials

**Figure 1:**
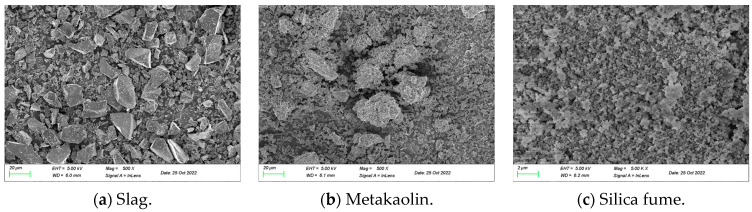
SEM images of raw materials of (**a**) slag, (**b**) metakaolin, and (**c**) silica fume.

Ground granulated blast furnace slag, metakaolin, and silica fume were adopted as precursors to prepare the geopolymer binder, and their chemical compositions and loss on ignitions (LOI) are listed in Table 1. In addition, the scanning electron microscope (SEM) images of the three precursors are also shown in Figure 1. Sodium hydroxide flakes with a purity of 96% and sodium silicate solution with a modulus ratio of 2.23 were adopted to prepare the alkaline activator solution. Natural river sand was selected as fine aggregates with a maximum particle size of 2.36 mm. The copper plated steel fiber with an average length of 14 mm and diameter of 0.2 mm was adopted, and the tensile strength and density were 2880 MPa and 7.85 g/cm^3^, respectively.

### 2.2. Mix Proportions

Nine groups of specimens were designed with different fiber dosages and sodium hydroxide concentrations to investigate the influence of steel fiber dosage and the molarity of sodium hydroxide solution on both compressive strengths and chloride penetration resistance. The detailed mixture proportions are provided in Table 2. As for the alkali activator solution, flake shaped sodium hydroxide solids were dissolved into deionized water to obtain sodium hydroxide solutions with a molarity (designated as M) of 4 mol/L, 8 mol/L, and 12 mol/L. After cooling for 24 h to reach an ambient temperature, the sodium hydroxide solution was then mixed with equal volume sodium silicate solution to obtain the alkali activator solution. In addition, steel fiber with three different volume fractions of 0%, 1%, and 2% were added into the matrix.

### 2.3. Specimens Preparations

The alkaline activator solution was prepared in advance and cooled for 24 h due to the exothermic effect when dissolving sodium hydroxide solids. The precursors, including ground granulated blast furnace slag, metakaolin, and silica fume, were mixed in a mixer for about 2 min. Subsequently, the river sands were added with the mixer continuously working for 1 min. The alkaline activator solution was then gently and uniformly poured into the mixture within 1 min and mixed for 3 min. Then, the steel fibers were added into the mixture and mixed for 2 min in order to obtain a uniform distribution. Finally, the mixture was poured into molds and covered with a plastic film to avoid moisture evaporation. The specimens were demolded after 24 h and cured in laboratory condition with a temperature of 20 ± 2 °C and a relative humidity of 72 ± 3%.

### 2.4. Testing Methods

The compressive strength of cubic specimens sized 70.7 mm × 70.7 mm × 70.7 mm were tested using the universal testing machine. The loading rate was set as 1 mm/min, and the averaged compressive strength of three specimens in each group was calculated.

**Figure 2:**
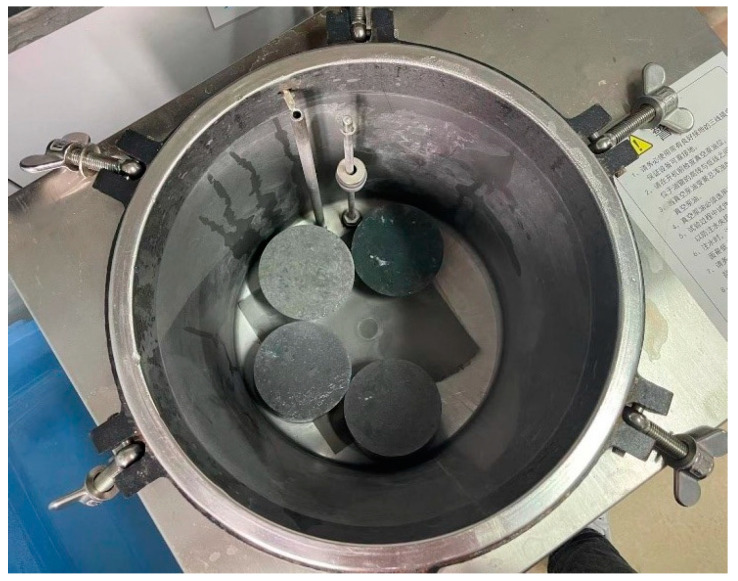
Specimens after vacuum saturation.

**Figure 3:**
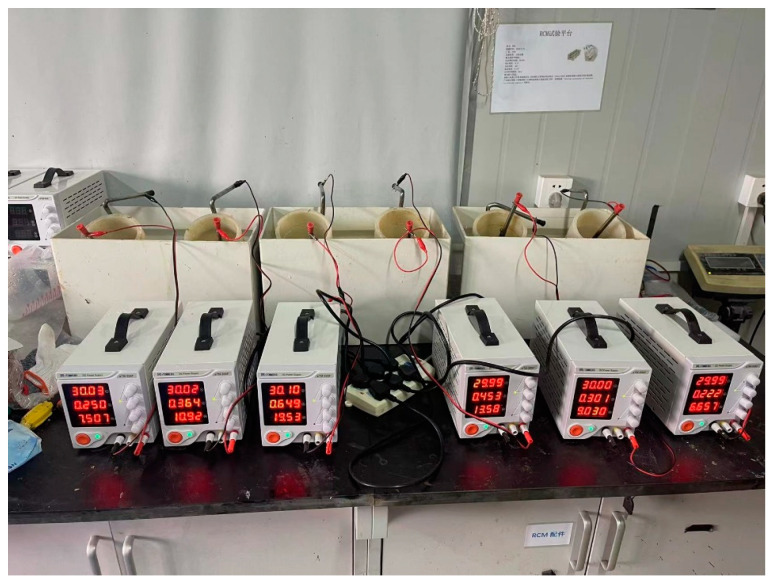
The experimental instrument used for the RCM test.

In addition, a RCM test was conducted following the NT Build 492 standard [35]. The cylindrical specimens with a diameter of 100 mm and a height of 200 mm were prepared and cut into four pieces with a thickness of 50 mm. Then, three samples with smooth cutting planes were adopted and vacuum saturated in advance, as shown in Figure 2. The cathodic electrolyte was 10% sodium chloride solution, and the anodic electrolyte was sodium hydroxide solution with a molarity of 0.3 mol/L, which were arranged in the axial direction of the samples. At first, a voltage of 30 V was axially applied across the specimens, and the current was recorded. Subsequently, the test voltage was adjusted to 10 V according to the observed current density to avoid a potential rise in temperature, and the power duration was 24 h. The instrument adopted for the RCM test is shown in Figure 3. Then, the specimens were split axially, and the cutting surface was sprayed with a 0.1 mol/L silver nitrate solution to determine the chloride penetration depth. The non-steady chloride migration coefficient was subsequently calculated using

(1) $$D_{nssm}=\frac{0.0239\left(273+T\right)L}{\left(U-2\right)t}\left(x_{d}-0.0238\sqrt{\frac{\left(273+T\right)L\cdot x_{d}}{U-2}}\right)$$

where *D_nssm_* signifies the non-steady chloride migration coefficient (×10^−12^ m^2^/s); *T* represents the average value of the initial and final electrolyte temperature (°C); *L* represents the thickness of the sample (mm); *U* represents the applied voltage (V); *t* represents the test duration in hours; and *x_d_* represents the average penetration depth of the chloride ions (mm).

## 3. Results and Discussions

### 3.1. Compression Test

#### 3.1.1. Failure Modes

**Figure 4:**
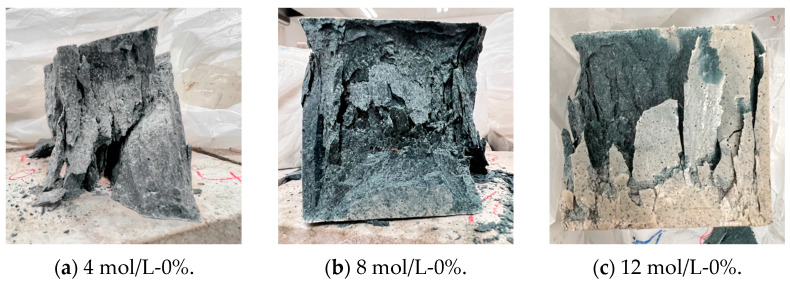

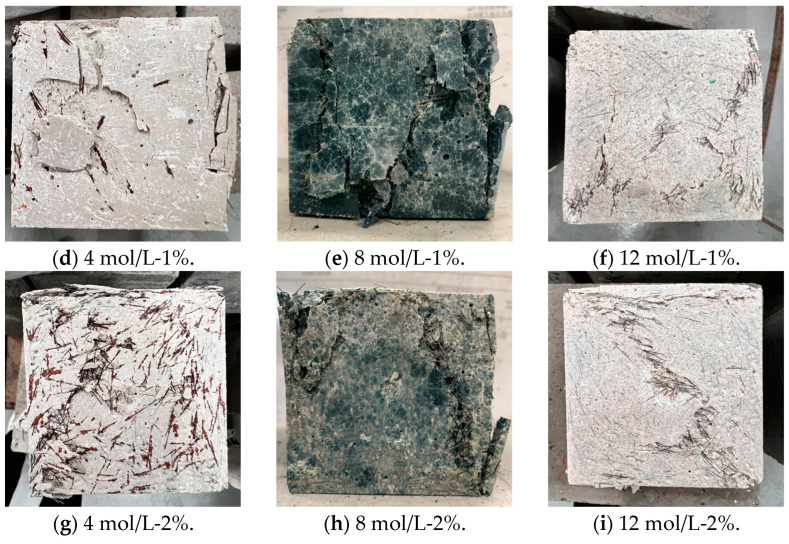
Typical failure modes under uniaxial compression.

The typical failure modes of the UHPGC specimens under the uniaxial compression test are shown in Figure 4. Figure 4a–c show that the integrity of the specimens without adding steel fibers was significantly improved due to the increase in the hydroxyl concentration of the alkali activator solution, and the core of the specimens still remained intact rather than cracking into pieces after reaching the peak load.

In addition, the failure modes were also different for the specimens produced using the alkali activator solution with the same molarity of sodium hydroxide (e.g., as shown in Figure 4a,d,g). It can be observed that the integrity of the specimens also increased with the increase in the steel fiber content in the concrete matrix, which was mainly due to the excellent bridging effect of the steel fibers, and the damaged parts that lost bearing capacity could still be attached to the main body. As can be seen from the figure, the steel fibers connected the concrete matrix on both sides of the crack; hence, they improved the load carrying capacity of the specimens. As shown in Figure 4g, the specimens made using the alkali activator solution with 4 mol/L NaOH solution also showed excellent integrity with a 2% steel fiber dosage, but surface peeling was also observed. However, for specimens with a 2% steel fiber dosage and 12 mol/L NaOH solution, as shown in Figure 4i, the specimen surface was still relatively flat, which again suggested the bridging effect of the steel fibers reinforcement.

#### 3.1.2. Influence of Fiber Dosage on Compressive Strength

**Figure 5:**
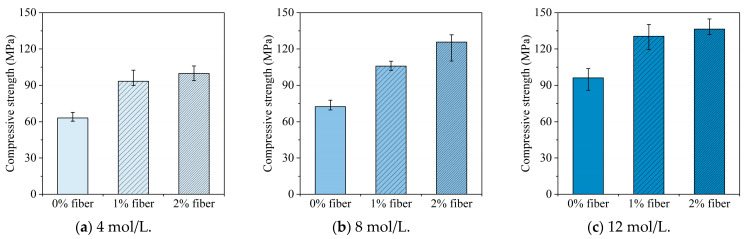
The influence of steel fiber dosage on compressive strength.

The compressive strengths of UHPGC for each group are listed in Table 3. In addition, the influence of steel fiber dosage on the compressive strengths of UHPGC with different NaOH solution concentrations is displayed in Figure 5 where the error bar characterizes the maximum and minimum compressive strengths of each group. Generally, it can be seen from the figure that the compressive strengths were all above 60 MPa and could maximumly reach 100 MPa for specimens without the reinforcement of steel fibers, which indicated the superior compressive performance of the proposed UHPGC design. In addition, adding steel fibers was beneficial as it improved compressive strengths. The most significant improvement in compressive strengths by adding steel fibers was observed when the 8 mol/L NaOH solution was adopted. Compared to these, without adding steel fibers, the compressive strengths increased 46.5% and 73.7%, respectively, for specimens with a 1% and 2% steel fiber dosage, which is shown in Figure 5b. On the contrary, the effect of the addition of steel fibers on compressive strengths improvement was less significant for specimens with 12 mol/L NaOH solution. As shown in Figure 5c, the compressive strengths increased by 35.9% and 41.8% when 1% and 2% steel fibers were added into the geopolymer matrix. This is because, on the one hand, the increase in the alkali concentration in the alkali activator solution could also lead to higher compressive strengths, which could make the contribution of steel fibers less significant. On the other hand, a higher NaOH solution concentration may result in an efflorescence effect and shorten the hardening time, so the dispersion of steel fibers in the geopolymer matrix could be less idealized and uniform, which could also limit the influence of the steel fiber content on the compressive strength improvement.

#### 3.1.3. Influence of NaOH Solution Concentration on Compressive Strength

**Figure 6:**
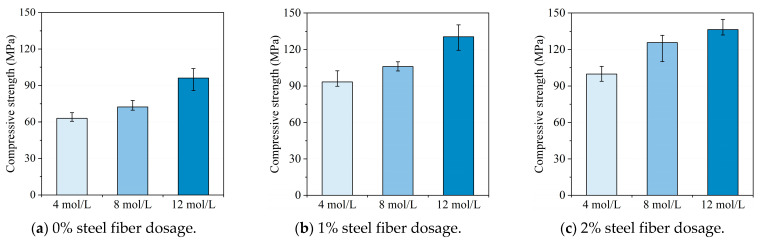
The influence of NaOH solution concentration on compressive strength.

The impact of sodium hydroxide molarity on the compressive strength of the slag/metakaolin-based UHPGC specimens with different fiber volume contents is illustrated in Figure 6. A general overview shows that the increase in sodium hydroxide molarity contributed to higher compressive strengths for specimens both with and without the addition of steel fibers. This can be attributed to the fact that the geopolymerization reaction can be intensified in a highly alkaline solution, which could increase the dissolving rate of silicate and aluminum phases into the solution and react to form gel. As a result, the higher NaOH solution concentration could lead to a refined pore structure of the UHPGC which is beneficial to the improvement in compressive strength.

The most obvious increase in compressive strength was observed in the case of 0% steel fiber dosage. Compared with UHPGC with 4 mol/L NaOH solution, the compressive strengths increased 14.58% and 52.3%, respectively, when the molarity of sodium hydroxide was 8 mol/L and 12 mol/L, respectively. This suggested that the impact of increasing NaOH concentration in the alkali activator solution was most significant for cases without steel fibers. Besides, for UHPGC with fiber dosages less than 2% (as shown in Figure 6a,b), the compressive strength improvement was much more significant between 8 mol/L and 12 mol/L (32.9% and 23.3%, respectively, for 0% and 1% steel fiber dosages) compared with the strength improvement between 4 mol/L and 8 mol/L (14.58% and 13.38%, respectively, for 0% and 1% steel fiber dosages). On the contrary, the compressive strength improvement between 8 mol/L and 12 mol/L was less obvious (only 8.6%) when 2% steel fibers were added, as can be seen in Figure 6c. This may be because the role of increasing NaOH solution concentration in compressive strength development tended to be less significant alongside the increase in steel fiber volume content.

#### 3.1.4. Combined Influence of Fiber Dosage and NaOH Solution Concentration

**Figure 7:**
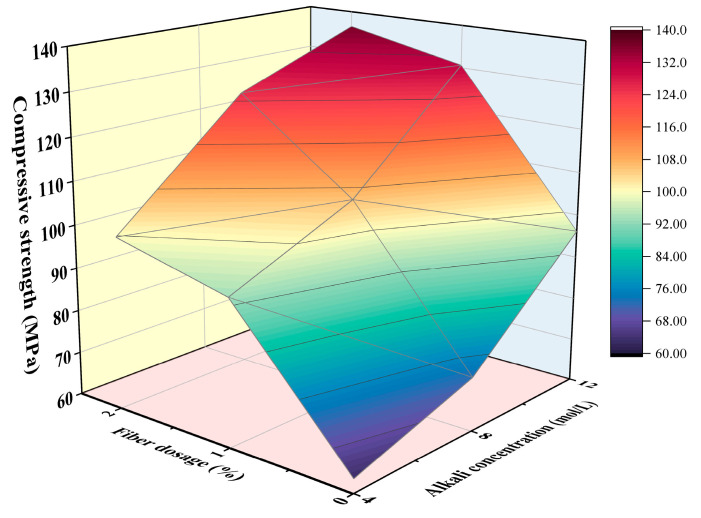
The combined influence of steel fiber dosage and NaOH solution concentration on the compressive strength of slag/metakaolin-based UHPGC.

According to the discussion regarding the influence of steel fiber dosage and alkali concentration, it can be found that both factors have a synergetic impact on the compressive strength, and each factor cannot be analyzed independently. Therefore, the combined influence of steel fiber dosage and sodium hydroxide molarity will be discussed in this section. Figure 7 displays the compressive strengths under fiber dosages ranging from 0% to 2% and NaOH solution concentration ranging from 4 mol/L to 12 mol/L. Generally, within certain ranges, both the increases in steel fiber dosage and NaOH solution concentration were beneficial for the improvement of compressive strengths. Meanwhile, it can also be observed that compared with the improvement amplitude with a higher NaOH solution concentration, adding more steel fiber dosages can result in a more obvious increase in compressive strength, while the increase degree was less significant with the increase in alkali concentration in the alkali activator solution.

Based on the experimental data, the compressive strength of slag/metakaolin-based UHPGC has been fitted as a function of steel fiber dosage and NaOH solution concentration using Equation (2) where *f_c_* signifies the compressive strength; *S* signifies the steel fiber volume fraction; and *A* represents the molarity of NaOH solution. R-square of the fitted equation was 0.9819, which suggested a good correlation between compressive strength with steel fiber dosage and NaOH solution concentration. However, it should be noted that the adopted experimental data was limited, to some extent, to obtain this fitted equation. Existing studies have reported that an excessively higher NaOH concentration in the alkali activator solution would conversely lead to a decreased compressive strength [36], and both fiber length and fiber types have a nonnegligible impact on mechanical properties [37]. Therefore, extensive results should be used to fit a more accurate empirical equation in a future study.

(2) $$f_{c}=49.85+42.07S+2.274A-11.06S^{2}+0.219A\cdot S+0.1224A^{2}$$

### 3.2. Rapid Chloride Migration Test

Chloride induced corrosion has always been a deleterious factor influencing the durability performance of concrete structures serving marine and saline environments [38,39]. The existence of chloride and the subsequently induced steel corrosion will severely limit the long-term performance of UHPGC for geopolymer concrete with steel fiber reinforcement. However, considering the limited literature that focuses on the chloride resistance capacity of slag/metakaolin-based geopolymer concrete, it is necessary to test the chloride penetration resistance of the fiber reinforced geopolymer concrete and further explore the underlying relationships between steel fiber content and alkali concentration with chloride penetration resistance. Therefore, a rapid chloride migration test was conducted in this study following the NT Build 492 standard, and the influence of steel fiber dosage and alkali concentration is discussed in the following subsections.

#### 3.2.1. Influence of Fiber Dosage on Chloride Penetration Resistance

The non-steady chloride migration coefficients measured using the RCM test are listed in Table 4. The non-steady chloride migration coefficient had a range from 6.5 × 10^−12^ m^2^/s to 14.1 × 10^−12^ m^2^/s. According to a recent study by Noushini et al. [40], chloride migration coefficients of fly ash based geopolymer concrete ranged between 38 × 10^−12^ m^2^/s and 79 × 10^−12^ m^2^/s, which were approximately 5 times higher than chloride migration coefficients of slag/metakaolin-based geopolymer concrete. The relatively lower chloride penetration resistance of the fly ash/slag-based geopolymer concrete may be caused by the reduced chloride binding capacity due to the addition of low calcium fly ash.

**Figure 8:**
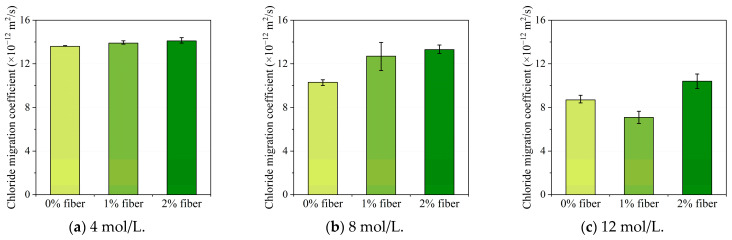
The influence of steel fiber dosage on chloride migration coefficient tested using RCM.

The impact of the steel fiber volume content in the geopolymer matrix is shown in Figure 8. It can be observed from Figure 8a,b that the chloride migration coefficient slightly increased with the addition of steel fiber content for geopolymer concrete with 4 mol/L and 8 mol/L NaOH solution, which suggested a relatively lower chloride penetration resistance. However, as shown in Figure 8c, the chloride penetration resistance firstly decreased with a 1% steel fiber dosage and then increased when 2% steel fiber content was adopted for geopolymer concrete activated with 12 mol/L sodium hydroxide solution. It should be noted that, according to an experimental measurement conducted by Vincler et al. [41], the addition of both steel fibers and polyvinyl alcohol fibers were both beneficial to improve the chloride penetration resistance of UHPGC samples, and corrosion was also found for samples with steel fibers. Therefore, in this study, considering the relatively lower pH value of the geopolymer concrete pore solution, the steel fibers in the UHPGC specimens with lower NaOH solution concentration would also experience corrosion during the RCM test procedure. Therefore, the formation of porous corrosion products on the vicinity of steel fibers could result in reduced chloride penetration resistance, which could also explain the slight increase in the chloride migration coefficient alongside the increase in steel fiber volume content. In addition, there would also be current enrichment and a rise in temperature during the test because of the steek fibers segregation, which could also lead to an increase in the chloride migration coefficient.

#### 3.2.2. Influence of NaOH Solution Concentration on Chloride Penetration Resistance

**Figure 9:**
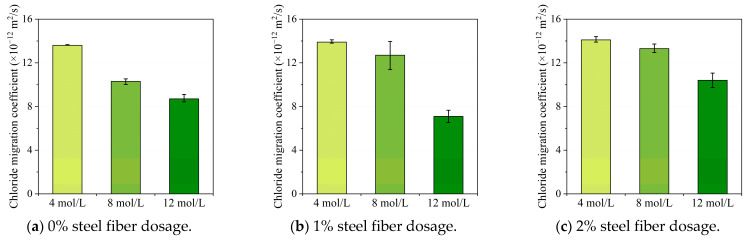
The influence of NaOH solution concentration on chloride migration coefficient tested using RCM.

Figure 9 illustrates the variation in chloride migration coefficients with different NaOH solution concentrations. Generally, it can be found from the figure that a higher sodium hydroxide concentration in the alkali activator solution was beneficial to improve the chloride penetration resistance of UHPGC specimens. In detail, when NaOH concentration increased from 4 mol/L to 12 mol/L, the chloride migration coefficient decreased 36.0%, 48.9%, and 26.2%, respectively. This could be explained considering the following two aspects. Firstly, an increase in the NaOH solution concentration could facilitate the dissolution of silicate and aluminum phases and contribute to a higher geopolymerization reaction degree. As a result, a denser pore structure in the geopolymer concrete will then lead to a better chloride penetration resistance in geopolymer concrete. In addition, since the corrosion of steel fibers would generate porous corrosion products and reduce the chloride penetration resistance, a higher NaOH solution concentration could reduce the corrosion risk of the steel fibers; hence, it could result in a relatively higher chloride penetration resistance.

**Figure 10:**
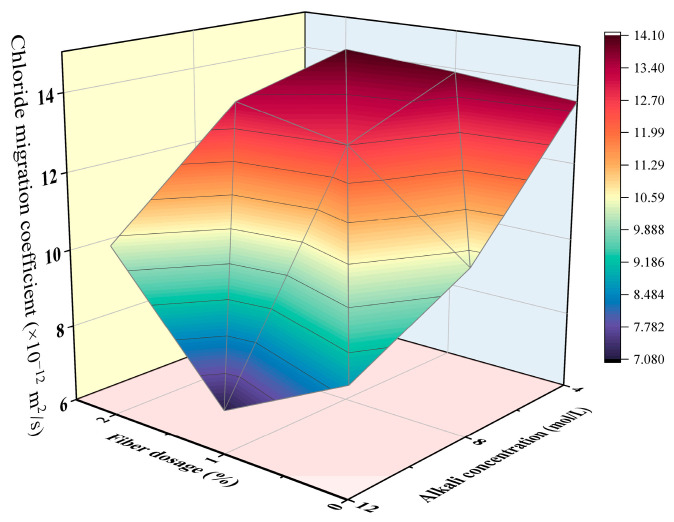
The combined influence of steel fiber dosage and NaOH solution concentration on the non-steady chloride diffusion coefficient.

The joint influence of steel fiber volume content and NaOH solution concentration on the non-steady chloride migration coefficient is plotted in Figure 10. As mentioned previously, except for the specimens with a 12 mol/L NaOH solution, the higher volume percentage of steel fibers contributed to the slightly higher chloride migration coefficient. As for the influence of the alkali concentration, the higher molarity of the NaOH solution concentration can lead to a reduced chloride migration coefficient for all fiber dosages, which suggested a better chloride penetration resistance.

It should be noted that NT Build 492 was originally proposed based on ordinary Portland cement concrete. The influence of pore solution pH on the color contour of white silver chloride depositions cannot be comprehensively considered for geopolymer concrete in which the chemistry of the pore solution would be different from ordinary Portland cement concrete [40]. In other words, when spraying silver nitrate on the splitting section, both white silver chloride depositions and brown silver oxide will be precipitated because of the reaction between silver ions with chloride ions and hydroxyl ions, respectively. The pH value of the pore solution would significantly influence the threshold point where the brown silver oxide depositions block the white silver chloride depositions [42]. The chemistry nature of geopolymer concrete pore solution is influenced by various factors, such as different types of raw materials and the water-binder ratio; therefore, the results from the RCM test might deviate from the real values. However, despite the inaccuracy introduced by the testing methods, the RCM test can still provide qualitative results about the variation in chloride penetration resistance for each specific mixture proportion where the chemistry of the pore solution is relatively stable.

## 4. Conclusions

In this study, the mechanical and durability properties of slag/metakaolin-based UHPGC were investigated by conducting uniaxial compression tests and rapid chloride migration tests. The influence of steel fiber dosage and NaOH concentration in alkali activator solution was analyzed. Based on the experimental results, the following conclusions can be made.

1. The addition of steel fibers can effectively limit cracks propagation because of the bridging effect. The maximum compressive strength of geopolymer concrete without steel fibers can reach 100 MPa, and adding steel fibers up to 2% can further increase the compressive strength to 140 MPa.
2. Increasing the molarity of alkali activator solution within the range of 12 mol/L can improve compressive strength due to the facilitated geopolymerization reaction. More significant improvement in compressive strength was observed when the sodium hydroxide concentration increased from 8 mol/L to 12 mol/L.
3. The non-steady chloride migration coefficient measured using the RCM test ranged from 6.5 × 10^−12^ m^2^/s to 14.1 × 10^−12^ m^2^/s. A higher steel fiber dosage slightly decreased the chloride penetration resistance, which is possibly attributed to steel fibers corrosion, current enrichment, and rising temperatures due to fiber segregation.
4. A higher alkali concentration in the alkali activator solution could significantly mitigate chloride penetration, which is mainly because the facilitated geopolymerization reaction in a higher alkali environment can result in a lower porosity and decrease chloride penetration depth.

## Figures and Tables

**Table 1:** Chemical compositions (wt.%) and loss on ignition (LOI) of the precursors.

	SiO_2_	Al_2_O_3_	CaO	Fe_2_O_3_	MgO	K_2_O	SO_3_	LOI
Metakaolin	57.22	38.98	0.92	0.73	0.26	0.23	0.18	1.39
Slag	30.37	15.08	42.19	0.27	8.03	0.56	2.11	0.85
Silica fume	98.23	0.27	0.28	0.06	-	0.14	0.95	0.06

**Table 2:** Mixture proportions.

Samples	Slag (kg/m^3^)	Metakaolin (kg/m^3^)	Silica Fume (kg/m^3^)	River Sand (kg/m^3^)	Waterglass (kg/m^3^)	NaOH Molarity (mol/L)	Steel Fiber (Vol. %)
M4-0%	641	321	107	388	279	4	0
M4-1%	641	321	107	388	279	4	1
M4-2%	641	321	107	388	279	4	2
M8-0%	641	321	107	388	279	8	0
M8-1%	641	321	107	388	279	8	1
M8-2%	641	321	107	388	279	8	2
M12-0%	641	321	107	388	279	12	0
M12-1%	641	321	107	388	279	12	1
M12-2%	641	321	107	388	279	12	2

**Table 3:** Average compressive strengths for each group of UHPGC.

Samples	M4-0%	M4-1%	M4-2%	M8-0%	M8-1%	M8-2%	M12-0%	M12-1%	M12-2%
Compressive strengths (MPa)	63.1	93.4	99.8	72.3	105.9	125.6	96.1	130. 6	136. 3

**Table 4:** Average non-steady chloride migration coefficients for each group of UHPGC.

Samples	M4-0%	M4-1%	M4-2%	M8-0%	M8-1%	M8-2%	M12-0%	M12-1%	M12-2%
chloride migration coefficients (×10^−12^ m^2^/s)	13.6	13.9	14.1	10.3	12.7	13.3	8.7	7.1	10.4

## Data Availability

Data supporting reported results can be found within the article.
